# Nonlinear relationship and threshold effect of D-dimer on preoperative deep vein thrombosis in patients with ankle fractures: a retrospective study

**DOI:** 10.3389/fsurg.2026.1856179

**Published:** 2026-07-15

**Authors:** Zi-Ruo Zhang, Pei-Pei Li, Dan Chen, Si Gao, Xing Fan, Hong-Mou Zhao, Jing Hu, Jin Tong, Hong Zhi

**Affiliations:** 1 Foot and Ankle Orthopedic Diagnosis and Treatment Center, Honghui Hospital, Xi’an Jiaotong University, Xi'an, Shaanxi, China; 2 General Medical Ward, Honghui Hospital, Xi’an Jiaotong University, Xi'an, Shaanxi, China; 3 Academic Development Department, Honghui Hospital, Xi’an Jiaotong University, Xi'an, Shaanxi, China; 4 Department of Nursing, Honghui Hospital, Xi’an Jiaotong University, Xi'an, Shaanxi, China

**Keywords:** ankle fracture, D-dimer, deep vein thrombosis, non-linear relationship, retrospective study

## Abstract

**Objective:**

The purpose of this study is to explore the association between D-dimer levels and the likelihood of preoperative deep vein thrombosis (DVT) in patients with ankle fractures.

**Methods:**

This retrospective study included ankle fracture patients admitted to Xi'an Honghui Hospital's Foot and Ankle Surgery Center from January 2024 to November 2025. Preoperative DVT was identified using Doppler ultrasound, and the relationship between D-dimer levels and DVT was analyzed using multivariate logistic regression and generalized additive models.

**Results:**

Among 818 patients, 13.45% developed preoperative DVT. D-dimer was an independent risk factor of preoperative DVT (OR = 1.14, 95% CI: 1.03–1.26, *P* = 0.010). There was a nonlinear relationship between D-dimer and DVT risk, After adjusting for confounding factors (age, sex, CCI, diabetes and preoperative waiting time),exploratory analysis identified an inflection point at 3.92 mg/L. Below this threshold, each 1 mg/L increase in D-dimer was associated with a significantly higher odds of DVT (OR = 1.60, 95% CI: 1.24–2.06, *P* < 0.001).

**Conclusion:**

D-dimer is independently associated with preoperative DVT in ankle fracture patients, and the relationship appears to be non-linear.

## Introduction

The ankle joint is an important structure of the human body. Ankle fractures make up 10.2% of all bone injuries and are frequently encountered (1, 2). Surgery affects ankle mechanics and function in the long term (3). Deep vein thrombosis (DVT) is a condition in which a blood clot typically forms in the veins of the leg, such as the femoral vein (4). DVT is a life-threatening complication after surgery and involves multiple genes and systems (5, 6). It can also cause limb swelling, pain, or fatal pulmonary embolism, thus worsening prognosis and postoperative recovery (7). Recognizing and preventing preoperative DVT early is essential for improving perioperative care in patients with ankle fractures. D-dimer, which results from the breakdown of cross-linked fibrin, features distinct connections between the D and E subunits and indicates an imbalance in blood coagulation and fibrinolysis (8). Earlier studies establish D-dimer as a standalone predictor of preoperative DVT in cases of traumatic fractures (9). However, studies specific to patients with ankle fractures are limited. These patients are a heterogeneous group with broad age variation (10). The highest injury rate is in 21–30-year-olds, followed by 51–60-year-olds, which differs from general traumatic fracture patients (11). Age differences cause variation in trauma mechanisms, fracture severity, and thrombosis pathology (12, 13). Furthermore, D-dimer, usually seen as a continuous variable, may not relate linearly to DVT risk. This is similar to the nonlinear relationship between hospital-to-surgery time, surgical delay, DVT (14, 15), and the threshold effect of hemoglobin on hospital stay (16). D-dimer's effect on DVT may also have a critical threshold. This research will explore the link between D-dimer levels and the risk of preoperative DVT in patients with ankle fractures. We hypothesize that this association is nonlinear and will identify the D-dimer threshold predicting increased DVT risk using threshold-effect analysis to better support clinical risk stratification and prevention.

## Methods

### Ethics approval and consent to participate

The study was approved by the Ethics Committee of Xi’an Honghui Hospital (acting as the Institutional Review Board) (Approval No. 2026-KY-052-01). This retrospective study utilized anonymized clinical data collected during routine care from January 2024 to November 2025. The ethics committee granted approval for the secondary analysis of these existing data prior to the commencement of data analysis. Due to the retrospective design and the use of de-identified data, the requirement for informed consent was waived. All procedures complied with the Declaration of Helsinki and the relevant national regulations for retrospective research.

### Participants

We included patients with ankle fractures diagnosed from medical records at the Foot and Ankle Surgery Centre, Xi'an Jiaotong University Affiliated Honghui Hospital. The study period was from 1 January 2024 to 1 November 2025. The original medical records provided demographic and clinical data. The criteria for inclusion were: (1) age ≥18 years; (2) having an ankle fracture verified by x-ray or CT scan; (3) receiving surgical treatment at this facility; (4) having clinical data accessible within the hospital. Exclusion criteria: (1) history of pulmonary embolism or DVT; (2) admission ≥48 h prior; (3) prior DVT investigation at another hospital before admission; (4) missing lower limb Doppler ultrasound results at our hospital; (5) prior antiplatelet or anticoagulant therapy for other conditions; (6) incomplete data.

### Hospital treatment

Upon admission, all patients began DVT prophylaxis using mechanical methods. The following day, each patient underwent serum D-dimer testing and lower-limb Doppler ultrasound. If no contraindications were present, subcutaneous low-molecular-weight heparin was also administered. Preoperative DVT diagnosis relied on Doppler ultrasound, with persistent intraluminal filling defects as the criterion.

### Variables

This research collects data on age, gender, fracture classification, hypertension, diabetes, coronary heart disease, age-adjusted Charlson Comorbidity Index (CCI), Body mass index (BMI), smoking status, presence of open fracture, admission mode, fracture side, preoperative wait time, and D-dimer levels. All enrolled patients uniformly received subcutaneous low-molecular-weight heparin on the day after admission, provided there were no contraindications. The study's dependent variable is the occurrence of DVT, detected via Doppler ultrasound.

### Statistics analysis

Continuous variables were first tested for normality using the Shapiro–Wilk test. Normally distributed data are presented as mean ± standard deviation (M ± SD), and non-normally distributed data as median with interquartile range (IQR). Categorical variables are presented as counts (percentages). Comparisons between the DVT and non-DVT groups were performed using the independent samples t-test for normally distributed continuous variables, the Mann–Whitney U test for non-normally distributed continuous variables, and the chi-square test for categorical variables. To examine the association between D-dimer levels and preoperative DVT, we performed binary logistic regression analyses. D-dimer was entered as a continuous variable, and all odds ratios (ORs) are reported per 1 mg/L increase. Three models were constructed: Model 1 (crude): unadjusted. Model 2 (minimally adjusted): adjusted for age, sex and BMI, as universally recognized confounders. Model 3 (fully adjusted): The covariates were preselected on the basis of clinical relevance and existing literature. To examine nonlinearity, a generalized additive model (GAM) was plotted, followed by two-piecewise logistic regression using the segmented package. Two analyses were conducted: unadjusted and adjusted for Model 3 covariates. A log-likelihood ratio test compared the linear vs. piecewise models. A two-tailed *P* < 0.05 was considered significant. Analyses used R 4.3.2.

## Results

### Patient characteristics

**Figure 1:**
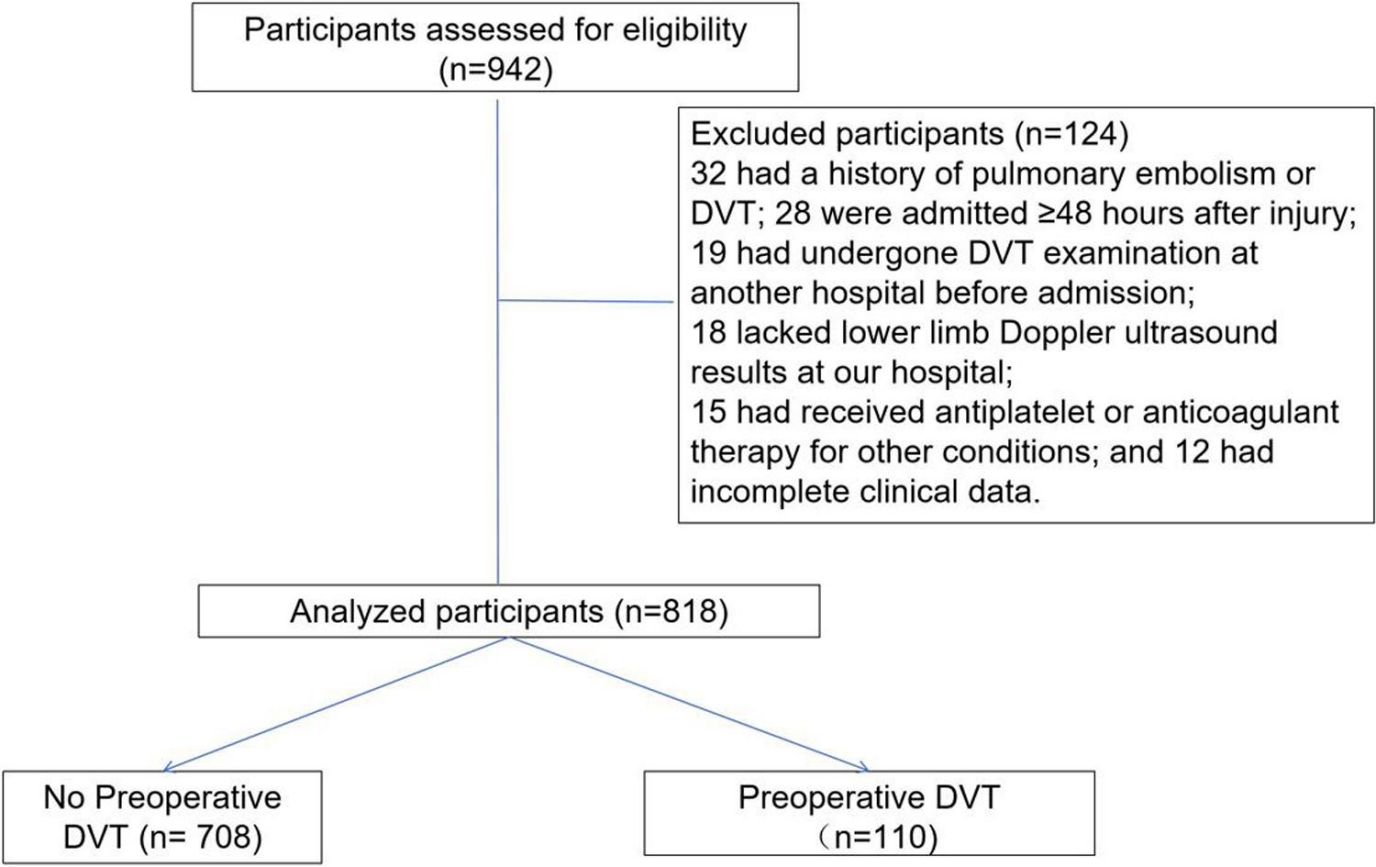
Flow chart summarizing the enrolment process.

A total of 942 patients with ankle fractures were initially screened. After excluding 124 patients based on predefined criteria, 818 patients were enrolled (Figure 1). Among them, 110 (13.45%) developed preoperative DVT in the lower limbs. Of these, proximal DVT involving the popliteal vein occurred in only 2 patients (1.8%), whereas the remaining 108 (98.2%) had distal calf DVT. Isolated muscular calf vein thrombosis (MCVT) was the predominant subtype, accounting for 88 patients (80.0% of all DVT cases). The other 22 patients (20.0%) had involvement of the deep axial calf veins (posterior tibial or peroneal veins).

Table 1 shows the demographic and clinical characteristics of the study population, comparing the non-DVT group to the DVT group. The DVT group had a mean age of (57.87 ± 12.15) years, while the non-DVT group had a mean age of (42.98 ± 16.07) years. The median CCI score was 2.00 in the DVT group, which was significantly higher than that in the non-DVT group (*P* < 0.001). Preoperative waiting time averaged 4.50 days in the DVT group and 3.00 days in the non-DVT group. Female patients constituted 62.73% of the DVT group and 49.72% of the non-DVT group. Patients with concomitant diabetes accounted for 12.73% of the DVT group and 4.80% of the non-DVT group. The two groups differed significantly in age, CCI, preoperative waiting time, D-dimer levels, diabetes mellitus, and gender (*P* < 0.05).

**Table 1:** Demographic and clinical characteristics of patients with and without preoperative DVT.

Variables	Preoperative DVT: No (*n* = 708)	Preoperative DVT: Yes (*n* = 110)	Statistic	*P*
Age (Mean ± SD)	42.98 ± 16.07	57.87 ± 12.15	*t* = −11.40	***P*** **<** **0.001**
BMI, M (Q₁, Q₃)	24.22 (23.34, 24.80)	24.22 (23.34, 25.38)	*Z* = −0.12	*P* = 0.903
CCI, M (Q_1_, Q_3_)	0.00 (0.00, 2.00)	2.00 (1.00, 3.00)	*Z* = −8.54	***P*** **<** **0.001**
Preoperative waiting time, M (Q_1_, Q_3_)	3.00 (2.00, 5.00)	4.50 (3.00, 6.00)	*Z* = −4.15	***P*** **<** **0.001**
D-dimer, M (Q_1_, Q_3_)	1.11 (0.72, 1.98)	2.21 (1.17, 3.72)	*Z* = −7.02	***P*** **<** **0.001**
Diabetes mellitus comorbidity, *n* (%)			*χ*^2^ = 10.83	***P*** **=** **0.001**
No	674 (95.20)	96 (87.27)		
Yes	34 (4.80)	14 (12.73)		
Coronary heart disease comorbidity, *n* (%)			*χ*^2^ = 3.08	*P* = 0.079
No	689 (97.32)	103 (93.64)		
Yes	19 (2.68)	7 (6.36)		
Fracture type, *n* (%)			*χ*^2^ = 5.17	*P* = 0.075
Triple ankle fracture	323 (45.62)	39 (35.45)		
Single ankle fracture	80 (11.30)	11 (10.00)		
Double ankle fracture	305 (43.08)	60 (54.55)		
Admission route, *n* (%)			*χ*^2^ = 0.93	*P* = 0.335
Emergency	87 (12.29)	10 (9.09)		
Outpatient	621 (87.71)	100 (90.91)		
Gender, *n* (%)			*χ*^2^ = 6.45	***P*** **=** **0.011**
Male	356 (50.28)	41 (37.27)		
Female	352 (49.72)	69 (62.73)		
Fracture side, *n* (%)			*χ*^2^ = 3.50	*P* = 0.173
Left	338 (47.74)	53 (48.18)		
Right	361 (50.99)	53 (48.18)		
Both sides	9 (1.27)	4 (3.64)		
Open fracture, *n* (%)			*χ*^2^ = 0.76	*P* = 0.383
No	690 (97.46)	105 (95.45)		
Yes	18 (2.54)	5 (4.55)		
Concurrent hypertension *n* (%)			*χ*^2^ = 2.24	*P* = 0.135
No	622 (87.85)	91 (82.73)		
Yes	86 (12.15)	19 (17.27)		
Smoking, *n* (%)			*χ*^2^ = 0.02	*P* = 0.879
No	588 (83.05)	92 (83.64)		
Yes	120 (16.95)	18 (16.36)		

t, t-test; Z, Mann–Whitney test; χ^2^, Chi-square test; SD, standard deviation; M, Median; Q₁, 1st Quartile; Q₃, 3st Quartile.

Bold values indicate statistical significance (*P* < 0.05).

### Multivariate analysis between D-dimer and DVT

We used binary logistic regression to examine the association between D-dimer (per 1 mg/L increase) and preoperative DVT(OR = 1.14, 95% CI: 1.03–1.26, *P* = 0.010), as detailed in Table 2. Both the unadjusted and adjusted models showed a significant association. In the crude model, D-dimer was associated with DVT (OR = 1.28, 95% CI: 1.18–1.40, *P* < 0.001). After adjusting for age and sex, the OR was 1.15 (95% CI: 1.04–1.26, *P* = 0.005). In the fully adjusted model (Table 2), the OR was 1.14 (95% CI: 1.03–1.26, *P* = 0.010).

**Table 2:** Results of binary logistic regression analysis for D-dimer and DVT.

Variables	*β*	S.E	*Z*	*P*	OR (95%CI)
Intercept	−6.39	0.72	−8.91	**<0**.**001**	0.00 (0.00∼0.01)
Diabetes mellitus comorbidity					
No					1.00 (Reference)
Yes	0.88	0.40	2.19	**0**.**028**	2.42 (1.10∼5.33)
Gender					
Male					1.00 (Reference)
Female	0.16	0.23	0.71	0.478	1.18 (0.75∼1.86)
Age	0.07	0.02	4.75	**<0**.**001**	1.08 (1.04∼1.11)
CCI	−0.20	0.14	−1.42	0.156	0.82 (0.62∼1.08)
D-dimer	0.13	0.05	2.58	**0**.**010**	1.14 (1.03∼1.26)
Preoperative waiting time	0.13	0.04	3.61	**<0**.**001**	1.14 (1.06∼1.23)

OR, odds ratio; CI, confidence interval.

Bold values indicate statistical significance (*P* < 0.05).

### Curve line correlation between D-dimer level and DVT risk

**Figure 2:**
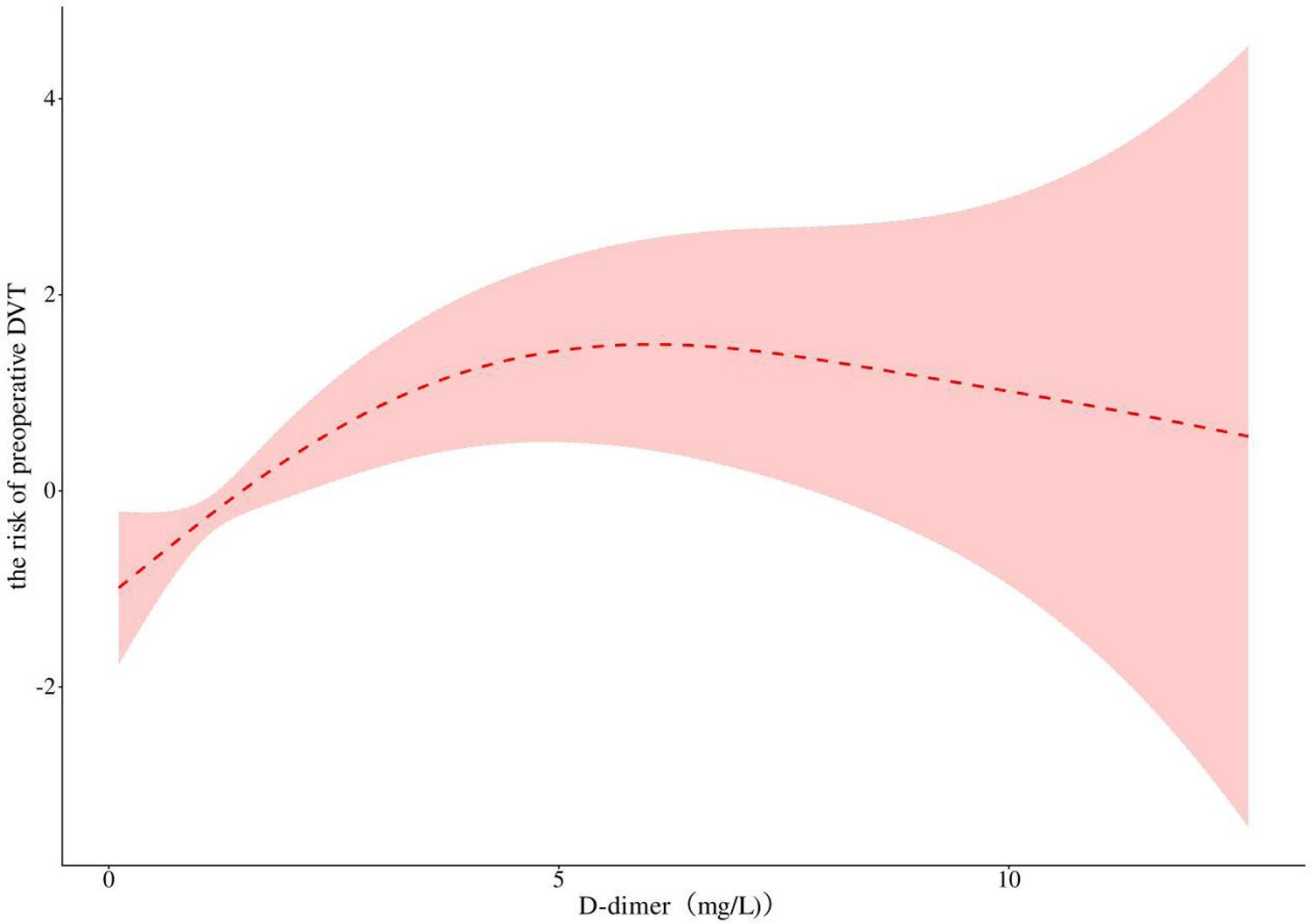
Graph depicting the fully adjusted dose–response relationship between D-dimer levels and the risk of preoperative DVT. The inflection point is a statistical estimate. External validation is required before clinical application.

As shown in Table 2, D-dimer was significantly associated with DVT risk overall. However, the log-likelihood ratio test comparing the linear model with a two-piecewise logistic regression model revealed a statistically significant threshold effect (*P* = 0.006; Table 3). The unadjusted model results are provided in the Supplementary Figure 1. The minimally adjusted model results are provided in Supplementary Figure 2 (adjusted for age, sex, and BMI).After full adjustment for age, sex, CCI, diabetes, and preoperative waiting time, the model remained stable. The fully adjusted dose–response relationship is illustrated in Figure 2, which displays the predicted probability of preoperative DVT (range 0–1) as a function of D-dimer level. The solid line represents the smoothed risk estimate derived from a generalized additive model, and the shaded area denotes the 95% confidence interval.

**Table 3:** The non-linear relationship between D-dimer and deep vein thrombosis after adjustment for confounding factors.

Outcome	Effect	*P*
Model 1 Fitting model by standard linear regression	1.14 (1.03–1.26)	**0**.**010**
Model 2 Fitting model by two-piecewise linear regression		
Inflection point	3.92	
< 3.92	1.60 (1.24–2.06)	**<0**.**001**
≥ 3.92	0.86 (0.65–1.14)	0.303
*P* for likelihood test		**0**.**006**

Bold values indicate statistical significance (*P* < 0.05).

As shown in Table 3, exploratory analysis identified an inflection point at 3.92 mg/L. Below this threshold, each 1 mg/L increase in D-dimer was associated with a significantly higher odds of DVT (*P* < 0.001).The distribution of D-dimer levels and DVT events according to this cutoff is presented in Table 4. In the subgroup with D-dimer < 3.92 mg/L, levels ranged from 0.11 to 3.89 mg/L (median, 1.10 mg/L; IQR, 1.08), with an observed DVT rate of 11.33% (84/741). Among patients with D-dimer ≥ 3.92 mg/L, levels ranged from 3.92 to 12.66 mg/L (median, 5.69 mg/L; IQR, 3.21), and the DVT rate was 33.76% (26/77). The OR for the upper segment was 0.86 (95% CI: 0.65–1.14).

**Table 4:** Distribution of D-dimer levels and DVT events according to the inflection point of 3.92 mg/L.

D-dimer group(mg/L)	*N*	DVT events	Median	Q1 (25th percentile)	Q3 (75th percentile)	IQR (interquartile range)
<3.92	741	84	1.10	0.73	1.81	1.08
≥3.92	77	26	5.69	4.71	7.92	3.21

## Discussion

This study found that the preoperative incidence of DVT in patients with ankle fractures was 13.45%, which is higher than that reported in studies on other foot and ankle conditions (17–19). This discrepancy stems in part from our exclusion of patients admitted ≥48 h after injury or those who had previously undergone DVT screening at other hospitals. Delayed admission or prior DVT screening at other facilities in these patients resulted in differences in baseline D-dimer levels and DVT risk, which may have confounded the observed association (15, 20). From an anatomical perspective, 98.2% of patients had distal calf DVT, which is higher than the 83.5% reported by Luo et al (17). This discrepancy stems from differences in preoperative waiting times: in Luo et al's study, most patients underwent surgery more than 24 h after admission, whereas the average preoperative waiting time in this study was 4.50 days. A longer preoperative waiting period may lead to increased immobilization of the lower limbs and worsened venous stasis, thereby promoting the formation and detection of DVT (21). Furthermore, isolated mid-calf VTE accounted for 80.0% of cases in this study, whereas most literature on ankle trauma does not report mid-calf VTE separately. However, recent perspectives suggest that mid-calf VTE may extend proximally or progress to symptomatic DVT, and its clinical significance warrants reevaluation (22).

The primary objective of this study was to investigate the relationship between preoperative D-dimer levels and the risk of DVT in patients with ankle fractures. Results from multivariate logistic regression analysis indicated that D-dimer is an independent predictor of preoperative DVT, a finding consistent with previous studies on hip fractures, tibial plateau fractures, and ankle fractures (17, 23, 24). Furthermore, this study found a significant nonlinear relationship and a threshold effect between D-dimer levels and DVT risk. After adjusting for confounding factors including age, sex, CCI, BMI, history of diabetes, and preoperative waiting time, the threshold inflection point was 3.92 mg/L. In the beginning stages of trauma, as tissue factor is released and inflammation heightens, the coagulation system becomes highly active, resulting in a quick surge in D-dimer levels, which is associated with an increased risk of DVT (25). However, when D-dimer levels were ≥ 3.92 mg/L, the two did not exhibit a statistically significant correlation. Within this high-value range, 26 cases of DVT were confirmed, which may be partially attributed to the small sample size and the broad confidence interval, resulting in limited statistical power.

D-dimer is a degradation product of cross-linked fibrin (8), but its relationship with thrombotic risk is not a simple linear increase. Similar findings have been reported in previous studies. Zhang et al. (25) found in patients with acute trauma that D-dimer levels were positively correlated with the severity of orthopedic trauma, suggesting that the interpretive context of D-dimer changes as the trauma burden increases. Chen et al (26) found a nonlinear dose-response relationship between D-dimer and DVT in patients with spontaneous intracerebral hemorrhage, with a statistical inflection point at 3.43 mg/L. The nature of the disease in that population is non-traumatic, whereas the ankle fracture patients included in this study were caused by direct or indirect trauma. Upon trauma, tissue damage, vascular endothelial disruption, and inflammatory responses can activate the coagulation cascade, promoting the formation of cross-linked fibrin. Concurrently, the fibrinolytic system is activated, leading to increased D-dimer production and a subsequent rise in plasma levels (27). Another retrospective study of patients with spinal cord injury also found a curvilinear relationship between age-adjusted D-dimer levels and DVT risk, with a statistical inflection point of 1.90 mg/L (28). This discrepancy stems not only from differences in the study populations but also from the selection of covariates. Zhang et al. sequentially adjusted for age, as well as age, sex, pulmonary infection, injury severity, ASIA classification, and occupation. Although this study adjusted for confounding factors such as age, sex, BMI, CCI, history of diabetes, and preoperative waiting time, several unmeasured variables may still influence the nonlinear relationship. This also suggests that the threshold inflection point identified in this study may be influenced by both included and unincluded confounding factors, necessitating a comprehensive assessment based on the patient's specific circumstances and local laboratory standards.

To date, few studies have examined the nonlinear relationship between D-dimer levels and DVT in patients with ankle fractures. Although this study identified a statistical inflection point in this population, its clinical applicability requires external validation. However, the finding of a nonlinear relationship itself offers new insights for thrombosis prevention and management: clinicians should not base decisions solely on absolute D-dimer levels but should incorporate other factors, such as age, comorbidities, and preoperative waiting time, into a comprehensive assessment.

This investigation also faces some constraints. Firstly, since it is a retrospective study from a single center, selection bias is unavoidable. External multicenter studies are necessary to confirm the general applicability of the conclusions. Although we adjusted for covariates preselected based on clinical literature, we were unable to obtain several potentially important variables due to the retrospective nature of the data, including: exact duration of limb immobilization before admission, detailed injury severity scores, ASA physical status classification, inflammatory biomarkers and the precise timing and dosage of anticoagulation. Thirdly, differences in D-dimer detection methods across institutions may require validation and calibration to local laboratory standards. Fourthly, Given that the inflection point of 3.92 mg/L was derived from exploratory analysis and the higher D-dimer segment comprised only 77 patients (9.41%), external validation in prospective studies is needed.

## Conclusion

D-dimer is independently associated with preoperative DVT in ankle fracture patients, and the relationship appears to be non-linear.

## Data Availability

The raw data supporting the conclusions of this article will be made available by the authors, without undue reservation.
