# Supplementary material for: Nonlinear relationship and threshold effect of D-dimer on preoperative deep vein thrombosis in patients with ankle fractures: a retrospective study
Source: Front Surg. 2026 Jul 15;13:1856179. doi: 10.3389/fsurg.2026.1856179 (PMC13412180; doi:10.3389/fsurg.2026.1856179)

Fig. 1. Graph depicting the unadjusted dose–response relationship between D-dimer levels and the risk of DVT

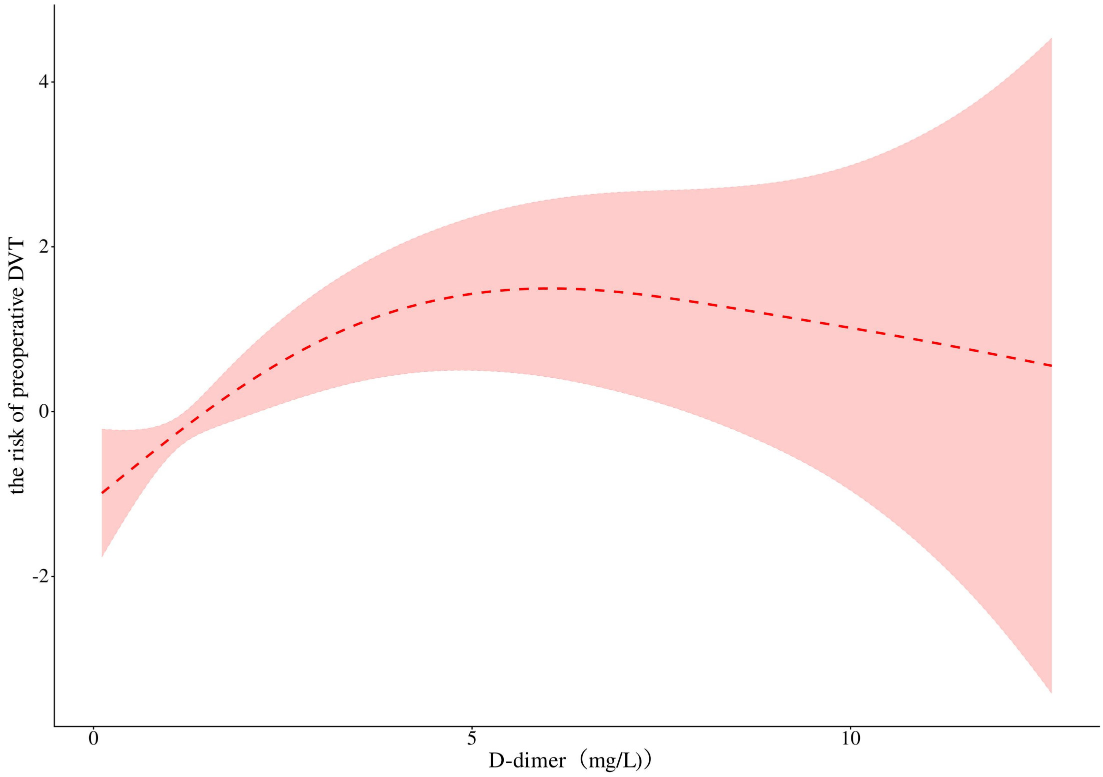

Fig2. Graph depicting the minimally adjusted dose−response relationship between D-dimer levels and the risk of preoperative DVT

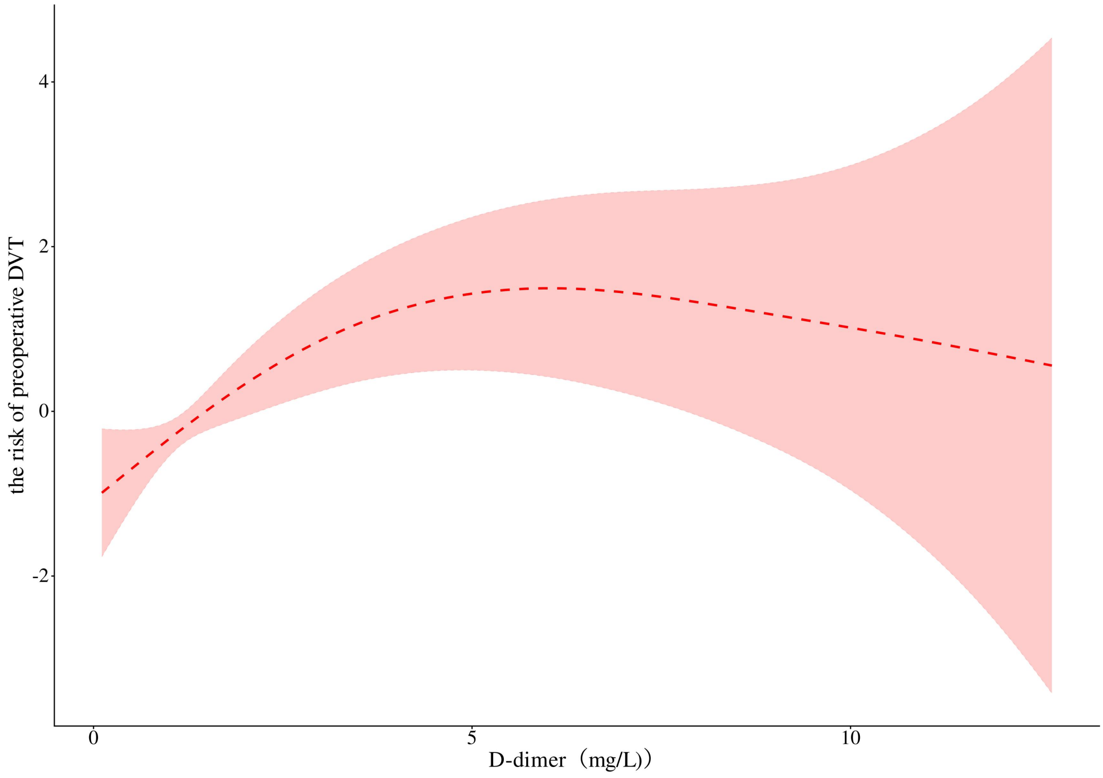

Supplement: Supplementary file 1 [file Datasheet1.pdf]
